# Is Animal-Assisted Therapy for Minimally Conscious State Beneficial? A Case Study

**DOI:** 10.3389/fpsyt.2020.00491

**Published:** 2020-05-28

**Authors:** Jacqueline P. Boitier, Marion Huber, Christian Saleh, Matthew J. Kerry, Margret Hund-Georgiadis, Karin Hediger

**Affiliations:** ^1^Department of Health, Zurich University of Applied Sciences (ZHAW), Winterthur, Switzerland; ^2^Department of Neurophysiology and Neurology, University Hospital Basel, Basel, Switzerland; ^3^Center for Neurorehabilitation and Paraplegiology, REHAB Basel, Basel, Switzerland; ^4^Clinical Psychology and Psychotherapy, Faculty of Psychology, University of Basel, Basel, Switzerland; ^5^Department of Epidemiology and Public Health, Swiss Tropical and Public Health Institute, Basel, Switzerland

**Keywords:** minimally conscious state, disorders of consciousness, animal-assisted therapy, human-animal interaction, behavior, neurorehabilitation

## Abstract

**Objective:**

The goal of this single case study was to qualitatively investigate the effects of animal-assisted therapy in a patient in a minimally conscious state.

**Method:**

We present a 28-year-old female patient in a minimally conscious state following polytrauma after a sports accident leading to cerebral fat embolism causing multiple CNS ischemic lesions. She received eight animal-assisted therapy sessions and eight paralleled control therapy sessions over 4 weeks. We investigated the reactions of the patient during these sessions *via* qualitative behavior analysis.

**Results:**

The patient showed a broader variability and higher quality of behavior during animal-assisted therapy compared to control therapy sessions.

**Conclusion:**

The observed behavioral changes showed higher arousal and increased awareness in the presence of an animal. The presented case supports the assumption that animal-assisted therapy can be a beneficial treatment approach for patients in a minimally conscious state.

## Introduction

Early rehabilitation is crucial for patients with disorders of consciousness (1). The term disorders of consciousness summarizes both vegetative state and minimally conscious state (MCS) (2). The vegetative state is defined as the first remission state after coma and is usually followed by the MCS. Bruno and colleagues (3) defined two stages of MCS: MCS minus (MCS−) and MCS plus (MCS+). Patients in MCS− show phases of wakefulness and basic communication skills, usually using some behavioral codes like eyelid shutting or finger movements. Additionally, the patients have the ability for object fixation with the eyes and the most can turn their eyes or their head to a stimulus. All these minimal reactions are shown inconsistently. The transition to MCS+ is characterized by consistent minimal reactions and additionally a consistent command following (3). Patients in a MCS+ state however, lack functional communication and/or do not demonstrate functional object use what is characterized as emergence from MCS (2).

The goal of therapeutic interventions for MCS patients is to extend the patient's wake phases, enhance the patient's consciousness, and stimulate complex behavior that provide learning possibilities *via* neuronal reorganization (4). Treatment concepts used for patients in neurorehabilitation are often activity-oriented and are highly related to everyday life. A series of studies have illustrated that emotionally relevant stimuli such as music or a familiar voice induce higher behavioral and cerebral response in patients with disorders of consciousness (5, 6), including animal visits (7). In the last years, a growing number of hospitals, rehabilitation centers and nursing facilities apply animal-assisted therapy (AAT) for patients with disorders of consciousness (8) while there is a lack of scientific research investigating this therapy approach. Animals seem to be emotionally relevant and engage attention of people more than objects do which is often discussed in the context of Wilson's Biophilia Hypothesis (9, 10). Borgi and Cirulli (10) demonstrate that animals have certain characteristics that elicit affectionate responses including readiness to care and social engagement. This illustrates why animals are seen as helpful partners within therapy for patients with disorders of consciousness. In a first controlled study, Hediger and colleagues showed beneficial effects of AAT in patients in a MCS compared to control therapy sessions (11). Beside such quantitative research on this topic it is crucial to learn for what patients AAT could be a promising approach and understand the individual reactions of patients. Therefore, we chose to combine our quantitative research design with a qualitative analysis.

In this case study, the aim was to qualitatively assess the effects of AAT in a patient in a MCS. We conducted a qualitative video-based behavior analysis of the patient in both animal-assisted and control therapy sessions.

## Materials and Methods

### Study Design

This exploratory, single case investigation was conducted as a part of a randomized, controlled within-subject study evaluating effects of AAT in patients in a MCS (1).

**Figure 1:**
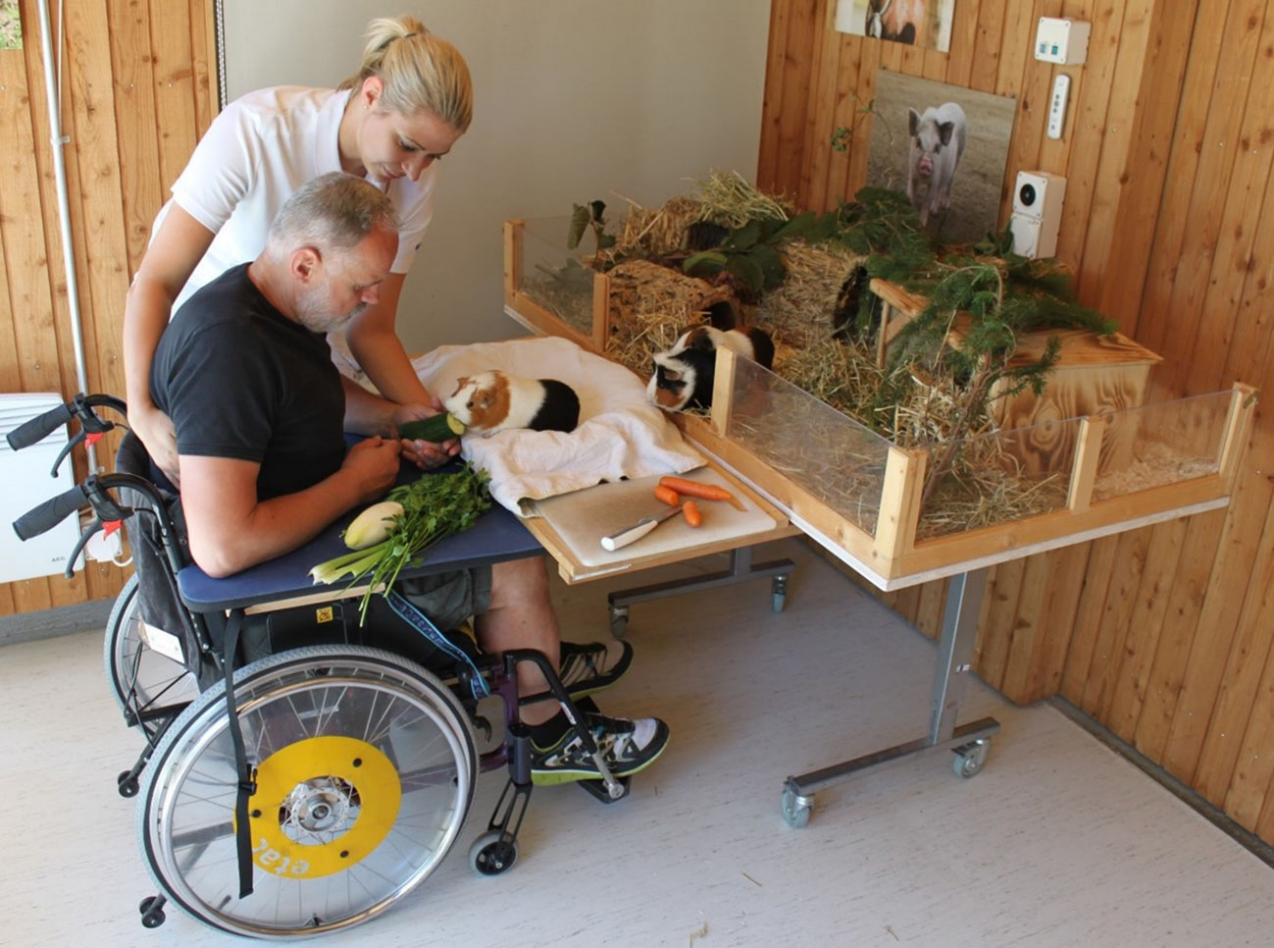
Exemplary setting of an animal-assisted therapy session with a patient in a minimally conscious state (a healthy volunteer posed in this picture to represent the setting). The figure has been published in Hediger (13).

The patient received eight therapy sessions with an animal (AAT) and eight control therapy sessions alternately over 4 weeks. Sessions were paralleled and matched as much as possible regarding therapeutic activities, the therapist, weekday and daytime. In this way, each two sessions were comparable with the exception of the presence of an animal in the AAT sessions. Each session included a beginning ritual and a closing ritual that consisted of sanitizing the patient's hands. **Table 1** gives an overview over the therapeutic activities in each analyzed session. The patient was mobilized into a wheelchair. To perform the activities, the patient's hands were physically guided [according to the Affolter^®^ concept (12), see **Figure 1**]. The sessions lasted between 10 and 19 min and took place in a designated room at a special therapy animal facility at a Swiss rehabilitation center. The study protocol was approved by the Ethics Committee for Northwest and Central Switzerland. The legal representative gave written informed consent for participation in the study and publication of the results.

**Table 1:** Content of the therapy sessions.

Session	Intervention	Content
1	AAT	Guinea pigs: preparing food, feeding, and stroking
2	Control	Touch fur and spiky massage ball
3	AAT	Guinea pigs: preparing food, feeding, and stroking
4	AAT	Guinea pigs: preparing food, feeding, and stroking
5	Control	Food preparation for animal: Cutting vegetables
6	Control	Food preparation for animal: Cutting vegetables
7	Control	Food preparation for animal: Cutting vegetables
8	AAT	Rabbit: preparing food, feeding, and stroking
9	AAT	Rabbit: preparing food, feeding, and stroking
10	Control	Food preparation for animal: Cutting vegetables
11	AAT	Rabbit: preparing food, feeding, and stroking
12	Control	Preparation of the animal's cage
13	Control	Food preparation for animal: Cutting vegetables
14	AAT	Guinea pigs: preparing food, feeding, and stroking

AAT, animal-assisted therapy.

All 16 therapy sessions were filmed and analyzed *via* a qualitative behavior analysis following a humanistic approach defined by Bernhard (14). This intensive procedure resulted in listing and categorizing behaviors of the patient as well as evaluating change over time in terms of quality of the behaviors. The categories were defined by the first author (JB) and validated by an expert (MH). The behavior was then assigned independently by JB and MH. In case of ambiguities, a consensus was reached after discussion.

### Patient Characteristics

**Figure 2:**
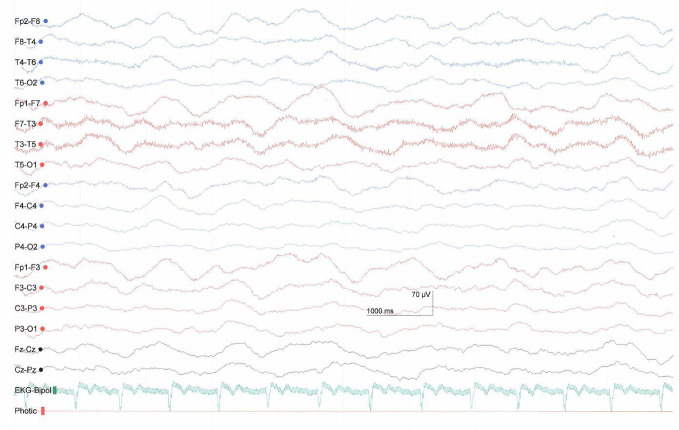
EEG one month before study start showing slow activity predominately in the delta-range.

The investigated patient was a 28-year-old female with a polytrauma after a sports accident leading to a cerebral fat embolism syndrome with several ischemic damages (supra- and infra-tentorial bihemisperic, basal ganglia/thalamus, and brainstem). The EEG showed slow-wave activity with predominantly delta waves (**Figure 2**). The patient was in early inpatient neurorehabilitation in a Swiss rehabilitation center and diagnosed with a MCS minus. She was enrolled into the study three months after the accident. On admission to the center her Glascow Coma Scale (GCS) score was 9 (eye opening = 4, verbal response = 2, motor response = 3, see **Supplementary Table 1**) and the original JFK Coma Recovery Scale (CRS) score was 9 (attention = 2, motor reaction = 2, reaction to auditory stimulus = 1, reaction to visual stimulus = 2, reaction to tactile stimuli = 1, oromotor reaction = 1, see **Supplementary Table 2**). When we started AAT, her GCS was 9 (eyes open = 4, verbal communication = 2, motor response = 3) and she had a CRS score of 15 (attention = 4, motor reaction = 4, reaction to auditory stimulus = 3, reaction to visual stimulus = 2, reaction to tactile stimuli = 1, oromotor reaction = 1, see **Supplementary Table 2**). Since the original CRS version does not include all of the behavioral criteria necessary to diagnose the MCS, diagnosis was based on clinical assessment by the responsible physician according to the Aspen diagnostic criteria (2) and to Bruno and colleagues (3) for the division of MCS+ and MCS−. Pharmacological treatment consisted of Catapresan (3 × 75 mcg per day), Lioresal (2 × 5 mg per day), Melatonin Suspension (1 × 2 ml), Nootropil (33%, 3 × 4.8 ml per day) *via* a PEG catheter. These doses remained unchanged during the study period. The patient received an individually targeted neurorehabilitation program consisting of five physiotherapy sessions, four occupational therapy sessions, five speech therapy sessions, one music therapy session and three neurofeedback sessions per week.

### Animal-Assisted Therapy

AAT sessions were based on the individual rehabilitation goals and consisted of basic activities selected from a range of occupational therapy assignments. The therapeutic activities were designed to have a connection with the animal present. For example, the task for the patient was to open a box with herbage and feed the herbage to the animal or to cut vegetables and feed the vegetables to the animal. The involved animals were three guinea pigs (5-year-old females) and a lop rabbit (castrated male, age unclear but older than 5 years). We chose to work with these species because they allow patients to observe them within a radius that is observable. These animals show interesting social behavior in groups, have a high interest in humans, and guinea pigs are also very communicative. They can be fed very easily but need mindful movements that they approach a human hand and therefore make it quite easy for the patient to recognize their emotional state. However, it must be noticed that both guinea pigs and rabbits need a special setting to work with patients with disorders of consciousness since they are highly prone to stress. The involved animals should have retreat possibilities at any time and be able to choose to interact with a patient or not to reduce possible stress (15). To ensure this, the animal keeper who knows the animals put them in a table cage with shelters and a platform in the front on which they could freely interact with the patient during the therapy sessions (see **Figure 1**). The animals' behavior was closely supervised by a study member and break-off criteria defined in a previous study (12) were in place. Since the animals were free to retreat into shelter whenever they wanted and were not forced to interact with the patient, no session had to be ended due to stress signals of the animals. All animals lived in a special enclosure in groups at the clinic and were familiar with interacting with patients with disorders of consciousness. The animals had regular veterinary checks and AAT as well as the handling and housing of the animals was conducted according to the guidelines of the International Association of Human Animal Interaction Organizations (16). The animal-related protocols were approved by the Veterinary Office of the canton Basel-Stadt, Switzerland.

## Results

The following behavior categories were identified in the explorative qualitative video analysis: changes in breathing; tone regulation; verbalization; and movements of the eye, gaze, mouth, chin, head, forehead, nose, and hand/arm. Over all, the patient showed an increased behavior diversity over the course of the 4 weeks, regardless of the intervention type. Additional to this change, there was also a clear difference in the patients' behavioral reactions between the two settings. The variability and the frequency of observed behaviors were higher in the AAT sessions compared to the control sessions. The patient had her eyes open during seven of eight AAT sessions, whereas she had the eyes open only during one of eight control sessions. We observed a clear increase in selective hand and finger movements over the 4 weeks which was more often observed in the AAT sessions. In the beginning of most AAT sessions the tone in the neck, the arms, and the hands and also the breath frequency increased. After a few minutes the patient showed a relaxation in the arms and hands. A tone normalization in the neck was observable nearly in each AAT session. With that, the patient was able to stabilize her head for some minutes by herself. In the control situations the head movements were less controlled and the head had to be held by an assistant most of the time.

We observed different persistent behaviors in relation to the context. The patient showed different behavioral reactions toward the different guinea pigs. She showed nose wrinkling and increased movements in both arms and hands toward one guinea pig with a tousled coat, but not toward the other two guinea pigs and the rabbit. Moreover, the patient turned her head away from one therapist persistently but not from others. The same behavioral sign was observed in the direct contact with the more tousled guinea pig. When the patient caressed the other guinea pig with the softer coat, the head turned to the middle and the nose wrinkling stopped. When the patient had herbs in her hands to feed the animals, tone regulation and selective movements in arm and the hand was observable. Behavioral signs as reactions to questions from the therapists were either head movements or nose wrinkling. When the therapist asked if either a spiky ball or a corncob would be suitable for the rabbit, the head turned to the corncob. The patient also showed increased movements with the arms or hands as reactions to questions. At the end of the 4 weeks, she showed attempts of verbalization.

## Discussion

The aim of the study was to explore and qualitatively assess the effect of AAT on behavioral signs of a patient in a MCS. Our results show that the patient showed broader variability of different behavioral reactions in the presence of an animal compared to control therapy sessions. Her reactions were also more consistent, shown with higher frequency, and had a higher quality during AAT sessions compared to control therapy sessions.

**Figure 3:**
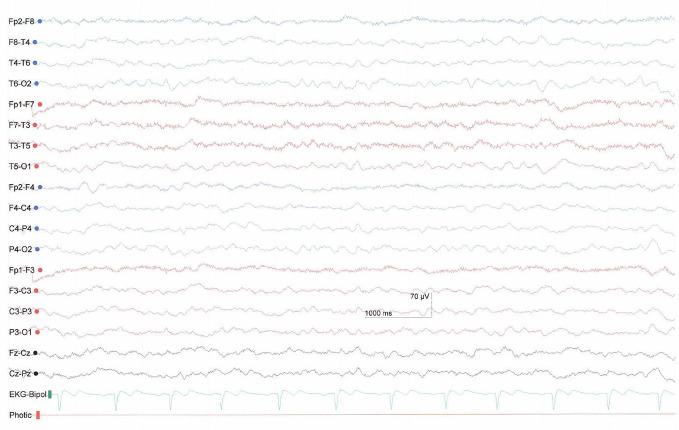
EEG two months after study end with predominantly theta activity showing slight improvement compared to the previous EEG.

The increase in movements and breath frequency during the AAT sessions in the present case study are interpreted as signs of higher arousal and increased awareness. Moreover, we interpreted some behaviors, such as head and finger movements, as signs of communication because they were seen more than one time over the different sessions and in relation to the context. The patient showed clear stimulus discrimination abilities. She could distinguish the three guinea pigs and we interpret that she had a favorite animal. The head turning in relation to one of the therapists and to one of the guinea pigs might be seen as a disapproval reaction. Also, a repeated EEG two months after the end of the study (**Figure 3**) showed a slight improvement in form of an increase in frequency predominately in the theta-range.

These results are in line with our trial that comprises quantitative data of the here presented patient. In this larger study, we found more behavioral reactions of MCS patients as well as an increased physical arousal measured *via* heart rate variability during AAT compared to control sessions (9). This leads to the conclusion that AAT might increase consciousness of patients. Another case report supports these findings. The authors investigated the reactions of a patient in a vegetative state to a dog and documented an increased wakefulness as well as more target-oriented movements over the course of the 54 sessions of animal-assisted intervention (17). The results are also in line with a recent study showing that dog-assisted therapy can facilitate emotional, social, and psychological goals in children and adolescents with severe neurological impairment (18).

We suggest that an animal can act as biographically and emotionally relevant stimulus for patients with disorders of consciousness, thus explaining the higher involvement during therapeutic sessions (5, 6). Moreover, AAT can be seen as a form of multimodal sensory stimulation (e.g., sound, touch, smell, vision), that contrasts the daily routine with animal presence and that exceeds basic forms of stimulation as already mentioned by Bardl and colleagues (14). Recent studies indicate that elaborate and emotionally relevant forms of stimulation have positive effects of the recovery process of patients with disorders of consciousness (19, 20) such as involving dynamic and naturalistic actions whereas basic forms of stimulation are considered unlikely to be effective (21). Moreover, animals provide a relevant form of social interaction and attachment (22), attract attention, and often lead to spontaneous affectionate responses (10). The patients can experience providing care for another living being, for example, by feeding a guinea pig against the fact that they are dependent from care themselves. This is a relevant role reversal and helps to cover basic human needs (23). Another important fact is that animals don't judge based on human categories (8). Animals communicate nonverbally—such as patients in a MCS—and provide physical proximity and tenderness that human caregivers cannot provide, but it must be beared in mind that rabbits and especially guinea pigs are not the species who should be petted (15). Other species like dogs are a better choice with regard to physical proximity. All these aspects describe innate mechanisms of forming relationships on an unconscious level which is crucial in a state of absent verbal language.

The results of this single case study cannot be generalized to other patients. Moreover, it is difficult to relate the behavioral improvements over the 4 weeks directly to the AAT intervention since the patient had also other complex therapy forms on a regular basis within the standard rehabilitation program or it could have been a spontaneous improvement. However, the increase in behavior variability and constancy were consistently linked to the AAT therapy sessions and were not observed in the control sessions. Another limiting factor is that the rater analyzing the videos could not be blinded to the condition since the animal was visible in the videos. Future research should explore mechanisms of AAT and investigate specific patient characteristics that define if AAT can be helpful, for example, if patients need to have a positive attitude toward animals. Moreover, effects of different animal species regarding different therapeutic goals should be investigated.

## Conclusion

Early interventions combined with a positive emotional involvement appear to be beneficial for patients with disorders of consciousness and have a broad impact on the subsequent rehabilitation process (15, 16, 24). Our results show that integrating an animal into a therapeutic activity can be a promising approach to foster neurorehabilitation for patients in a MCS.

## Data Availability Statement

The datasets for this article are not publicly available because it consists video material of the patient. Requests to access the transcribed data of the qualitative behavior analysis should be directed to KH, karin.hediger@unibas.ch. The transcription will be made available by the authors, without undue reservation, to any qualified researcher.

## Ethics Statement

The studies involving human participants were reviewed and approved by Ethics Committee for Northwest and Central Switzerland. Written informed consent was not provided because informed consent was provided by the legal representative and not the patient herself because she was in a minimally conscious state. The patient's legal representative provided written informed consent to publish the case report. Written informed consent was obtained from the individuals for the publication of any potentially identifiable images or data included in this article.

## Author Contributions

KH and MH had the idea for the study and KH and MH-G designed the study. KH contributed to the data acquisition. JB carried out the analysis. JB, KH, CS, MH and MK wrote the first draft of the manuscript. All the authors contributed to and have approved the final manuscript.

## Funding

KH currently receives an Ambizione grant (grant number PZ00P1_174082/1) from the Swiss National Science Foundation and funding from the Förderverein pro REHAB Basel. Further, this work was supported by the institute of Zurich University of Applied Sciences.

## Conflict of Interest

The authors declare that the research was conducted in the absence of any commercial or financial relationships that could be construed as a potential conflict of interest.
