# Supplementary material for: Is Animal-Assisted Therapy for Minimally Conscious State Beneficial? A Case Study
Source: Front Psychiatry. 2020 May 28;11:491. doi: 10.3389/fpsyt.2020.00491 (PMC7270332; doi:10.3389/fpsyt.2020.00491)
Supplement: Supplementary file 1 [file DataSheet_1.docx]

**Supplementary Table 1.** Scores and corresponding behavior on the Glascow Coma Scale (GCS).

| **Scale** | **Score** | **Criterion** | **Rating** |
| --- | --- | --- | --- |
| Eye opening | 4 | Open before stimulus | Spontaneous |
| Verbal response | 2 | Only moans / groans | Sounds |
| Best motor response | 3 | Bends arm at elbow, features clearly predominantly abnormal | Abnormal flexion |

**Supplementary Table 2.** Scores and corresponding behavior on the original JFK Coma Recovery Scale (CSR).

| **Scale** | **Timepoint** | **Score** | **Criterion** |
| --- | --- | --- | --- |
| Attention | On admission | 2 | Eyes open spontaneous |
|  | Before AAT | 4 | Stays with the stimulus (for longer than 5 minutes) |
| Motor reaction | On admission | 2 | Decorticate response |
|  | Before AAT | 4 | Normal posture reaction observable |
| Reaction to auditory stimulus | On admission | 1 | Vegetative (fright) reaction (startle) |
|  | Before AAT | 3 | Recognizes familiar music, voices etc. |
| Reaction to visual stimulus | On admission | 2 | Fixates pictures, persons or objects |
|  | Before AAT | 2 | Fixates pictures, persons or objects |
| Reaction to tactile stimuli | On admission | 1 | Vegetative reaction |
|  | Before AAT | 1 | Vegetative reaction |
| Oromotor reaction | On admission | 1 | Moans, groans |
|  | Before AAT | 1 | Moans, groans |
